# Fishermen do more than fish: local ecological knowledge of raftsmen about the arboreal species used to construct rafts (Bahia, Brazil)

**DOI:** 10.1186/s13002-018-0279-7

**Published:** 2018-12-27

**Authors:** Isis Leite Medeiros Mascarenhas Andrade, Marcelo Schramm Mielke, Nivaldo Peroni, Alexandre Schiavetti

**Affiliations:** 1 Programa de pós-graduação em Desenvolvimento e Meio Ambiente—PRODEMA (Master Degree Program in Development and Environment), Universidade Estadual de Santa Cruz (Santa Cruz State University), Ilheús, Bahia Brazil; 2 Departamento de Ciências Biológicas (Department of Biological Sciences), Universidade Estadual de Santa Cruz (Santa Cruz State University), Ilhéus, Bahia Brazil; 3 Laboratório de Ecologia Humana Etnobotânica (ECOHE) (Laboratory of Human Ecology), Departamento de Ecologia e Zoologia (ECZ), Universidade Federal de Santa Catarina (UFSC) (Santa Catarina Federal University), Florianópolis, Santa Catarina Brazil; 4 Investigador Asociado CESIMAR, CE- NPAT, Puerto Madryn, Argentina

**Keywords:** Ethnobotanics, Pau de Jangada, Traditional raft, Atlantic forest

## Abstract

**Background:**

Traditional raft (*jangada*), *piúba* wood raft (*jangada de pau de piúba*), six-log raft (*jangada de seis paus*), and wooden raft (*jangada de pau*) are some of the names given to the traditional Brazilian watercrafts created from the buoyancy of bound logs. The traditional raft is a watercraft used and built by artisan fishermen who have, throughout generations, kept and improved knowledge related to this practice and the use of the plant species they need as raw materials. Active groups of these fishermen and their watercrafts are distributed along 200 km of the coast of the state of Bahia. The fishermen interviewed in this study are at the southern limit of distribution for the use of this type of vessel.

**Methods:**

This study aimed to characterize the use of the arboreal species applied in the construction of the traditional raft in the municipalities of Uruçuca, Ilhéus, and Canavieiras in the southern State of Bahia, Brazil. For this purpose, structured and semi-structured interviews were individually conducted with 36 fishermen, and walking tours were conducted with specialists in the construction of the watercraft.

**Results:**

We observed that the raftsmen use 21 species to construct the traditional raft. The features of the wood, such as density, flexibility, and availability, are the main criteria applied to choose the arboreal species. Some species are preferred, such as *pau de jangada* (*Apeiba tibourbou*) and *biriba* (*Eschweilera ovata*), which are the most frequently employed in watercraft manufacturing.

**Conclusions:**

The southern Bahia population is familiar with the different tree species that are linked to their fishing activities. The main link between the fishermen and the useful species is present in the practice of raft construction. Currently, the restricted access to raw materials limits this practice, which consequently results in the cultural erosion of this community.

## Introduction

The ecological knowledge of artisanal fishers about the environment in which they fish is already well documented. However, this knowledge goes beyond fishing activity. It is a knowledge that accesses information from various components of the ecosystem, including the species used in the construction of fishing technologies, such as materials used for the manufacture of tools and the construction of vessels [1–3].

The production of fishing technologies relies on the choice of the plant species adapted to the ecological and cultural conditions related to fishing. The abilities of the fishermen to produce this technology come from the successful capture of fish through an optimized choice of the utilized plant species [3, 4].

Several ethnobotanical studies were conducted in artisanal fishing communities in Brazil and worldwide. Many of them aimed to analyze the plant usage by these communities [5–8], while others focused on the use of plants with medicinal purposes [9, 10], and some addressed studies of plants utilized for the construction of vessels and fishing tools in communities [2, 11–13]. These studies present a high interdependence between the knowledge and usage of terrestrial and marine biodiversity [1, 7, 14, 15].

The Brazilian maritime patrimony is formed by a rich set of traditional watercrafts that belong to the history and the landscape of the country, representing the geographical specificities according to the historical, environmental, and cultural features. For example, the raft (*jangada*), found in Northeast Brazil, is characterized by the junction of many logs. Similarly, the *tolda* canoes are present in the São Francisco River, which is located between the northeast and southeast regions of Brazil [16]. Relatively to the large number of watercrafts and the cultural richness that these boats represent, few ethnobotanical studies have been conducted that aim to characterize how plant resources are used in the construction of these traditional watercrafts and in fishing activities [17–19].

The traditional raft (*jangada*), *piúba* wood raft (*jangada de pau de piúba*), six-log raft (*jangada de seis paus*), and wooden raft (*jangada de pau*) are some of the names given to the watercraft created from a construction that ensures the buoyancy of many wooden logs joined together [20]. These watercrafts are considered to be adapted to the environment and the fishing style, and they are formed by the hull, mast, and support devices. The construction is handcrafted by fishermen using fittings and ties and without the need for nails, screws, or any other hardware [20]. Although the traditional raft has disappeared in many locations on the northeast shore where they were common in the past [21], there are active spots where the traditional rafts are still used and built by raftsmen. The most active region is located in a strip approximately 200 km south of Bahia State [19–22]. Along with this shore strip, there are semi-desert beaches rounded by the Atlantic Forest, where arboreal species are used as plant resources necessary for the construction and maintenance of the rafts [23–25].

With an understanding of the cultural importance of the raft as a traditional watercraft used by a group of experienced fishermen, this study aimed to evaluate how arboreal species are used in the construction of traditional rafts by groups of raftsmen settled in the southern region of the state of Bahia. We expect that our results may be applied to the development of conservation strategies and actions that aim to preserve the Atlantic Forest and traditional fishing. Such strategies should also consider the impact of the human groups who know the biome’s importance for the development of their daily activities.

## Materials and methods

### Study area

The state of Bahia has the largest seacoast in Brazil with 1188 km of continuous shore. Along with this coast, there are 44 municipalities and approximately 350 fishing communities. The state’s fishing fleet is mostly formed by non-motorized vessels [26].

**Fig. 1:**
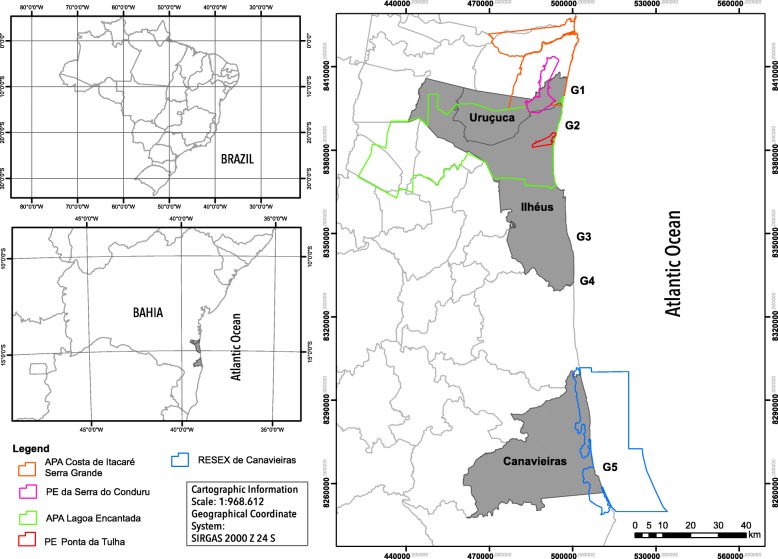
Map of the coastal strip of South of Bahia where the study was conducted

Fishermen who use traditional rafts are distributed along 200 km of the coast of the state of Bahia. The fishermen interviewed for this study were in the southernmost part of the Brazilian Northeast region. The raft is also used by small groups of fishermen on the north boundary of Bahia within the state of Sergipe [21] (Fig. 1).

Groups of fishermen from the southern part of the Northeast region are located in the surroundings of the Atlantic Forest, a biome that is an international hotspot and a priority area for conservation due to the extinction of a large part of the plant cover in an area with a high level of endemism and biological richness [27–29].

The study area comprised the municipalities of Canavieiras, Ilhéus, and Uruçuca (Fig. 1). These towns are located in the southern part of the state of Bahia, a region that has a hot and humid tropical climate without a dry season, with over 1300 mm of precipitation/year and with the most intense rainy period occurring between March and September [30].

### Raftsmen

We considered all artisan fishermen who use the traditional raft as a watercraft as raftsmen when we conducted our research. Among them, we were able to identify the raftsmen who mastered the techniques to construct the rafts when the individual interviews were conducted.

The total population of fishermen who used the rafts as fishing vessels was 46 raftsmen, among whom 36 agreed to participate in the research. The interviewed fishermen were placed in five groups according to their geographic location in the studied municipalities (Fig. 1).

Then, we characterized the groups by examining the number of raftsmen per group, the location, the proximity to protected areas [31, 32], and the width of the continental platform, which is the distance of the seabed from the continental platform at the spots where the rafts were found [33]. The fishing strategy, the type of raft used, and the distance traveled by the raft are presented in Table 1.

**Table 1:** Numerical and spatial characterization of the raftsmen studied groups, Southern Bahia, Brazil

Groups of raftsmen	Group 1	Group 2	Group 3	Group 4	Group 5
Number of raftsmen	26	6	3	5	6
Number of participants	23	4	3	1	5
County	Uruçuca	Ilhéus	Ilhéus	Ilhéus	Canavieiras
Location (collect point)	Serra Grande	Mamoã	Olivença	Acuípe	Atalaia
Continental shelf width	Short (5 km)	Short (5 km)	Large (20 km)	Large (20 km)	Large
Protected areas (UICN category/number)	II—01 V—02	II—01 V—01	**	**	VI—01
Fishing strategy	Line	Line	Trawls	Trawls	Trawls/line
Raft type	Motor and sail	Motor sail	Not sail	Not sail	Sail
Average distance traveled	2.00	2.10	0.30	0.15	1.30

*It features the distance reached on the ocean floor from the continental shelf where the rafts were found [33]

**Data not available

### Ethnobotanical research

Data were collected from March to December 2015 by the individual interview method [34, 35]. All interviews were recorded with audio at the time and place set by the interviewees. The interviews had an average duration of 50 min. The interviews were conducted in semi-structured and structured forms. In the structured interview, all interviewees responded to a set of stimuli that were as identical as possible through the use of a questionnaire, a free listing, and a visual stimulus [34, 35].

The information obtained was complemented and enriched by using ‘walking interviews’ [36] to avoid errors in species identification, as the raftsmen pointed to the species mentioned in loco [37]. Samples of the specimens were collected and later herborized, identified, and incorporated into the HUESC herbarium collection, and duplicates were sent to the CEPEC herbarium. The identification of the species was conducted by consulting specialized literature, by the supervision of a botanist and a para-botanist, by comparing specimens from national and international herbaria with a high-resolution image available online [38, 39], and through field observations. Next, we examined the association between the uses of the species and their applications available in the specialized literature. Plants were classified according to their origin: native (originally from Brazil) and exotic (introduced from another country) based on the List of Species of the Brazilian Flora [40].

Data collection was conducted in three stages: (1) identification of the area and population, in which we characterized the traditional rafts by visiting the communities where the raftsmen live and (2) execution of the semi-structured and structured interviews to determine socioeconomic aspects through the questionnaire and to collect information about the knowledge related to the traditional raft and the fishing activity through a guided semi-structured interview. During the interviews, we used free listing to identify the tree species useful in the construction of the traditional raft. After the listing was asked of each interviewee, the reason for choosing the tree species used in raft construction was determined. To assist this process and to have all parts of the raft identified, visual stimuli through photographs of all raft structures were used. (3) The walking interviews that occurred in parallel with the second stage were performed only with the raftsmen who mastered the construction of the raft and who were able to participate in this stage of the research. At that time, we collected fertile specimens (with flower and fruit) for identification and herborization.

### Data analysis

We measured and tabulated the height, width, length, and diameter of each raft that was found to elaborate technical drawings of the models. Data related to the characterization of the interviewees were treated using descriptive statistics.

Data were qualitatively classified through an ethnobotanical inventory confirmed with the register of the mentioned species (folk and taxonomic names) containing family, scientific name, popular name, origin, uses, and parts used [34]. The species referred to as useful for the production of the traditional raft were associated with each raft component. With the use of descriptive statistics, we calculated the average number of mentions of the valuable species to the production of each component of the raft, according to the calculation:

$$ \boldsymbol{M}=\left(\sum X\right)/n $$

where *M* is the average number of mentioned uses of the component, *X* is the number of times that the species was mentioned as being useful for that component, and *N* is the total number of species used for the production of the component.

Therefore, species with more remarks than the average for one component were considered as preferential for the production of this particular raft element. This calculation was done to determine the preferred species for the set of interviewees and was separately applied to groups with a sample more significant than one raftsman (groups 1, 2, 3, and 5) to determine the preferential species for each group.

The arboreal species mentioned were quantitatively evaluated by the use value (UV). The UV [41] is represented by the number of uses the species has and is calculated by dividing the sum of mentions of use for a determined species by the total number of informants. The equation, as suggested by Rossato et al. [42], is

$$ \mathbf{UV}=\left(\sum \mathrm{Us}\right)/N, $$

where UV is the use value of the species, Us is the number of mentioned uses by each informant for the species, and *N* is the total number of informants.

For species used to build the rafts, we calculated the specific UV according to the use of the species in the construction of the watercraft. For that purpose, the use was associated with the presence of the species in the different components of the traditional raft. For example, knowing how many different manufacturing components the mentioned species is used for is useful.

In addition to the UV calculation, we also calculated the Index of Cultural Significance (ICS), as established by Turner [43], to numerically express the role of plants in a culture. This index is calculated by a score given by the researcher leading to the species value. The formula adopted in this research was adapted by Silva et al. [44], where the values given to the variables (*i*, *e*, and *c*) are 2 or 1 for each mention of the use of each species. This adaptation gives the formula a more objective character:

$$ \mathrm{ISC}=\sum \left(i\times e\times c\right)\ \mathrm{FC}, $$

where *i* considers the impact of the plant in the daily life of the community; the value 2 is given to species that are grown, managed or manipulated, and the value 1 is given to the species found in the area that are still free from any management or conservation practices; and *e* is the preference of the use of a species compared to some other uses for a determined function. The value 2 is suggested for a species that is preferably used for a specific purpose, and the value 1 is suggested for other available species that are non-preferential for this purpose; c is the frequency of use. The value 2 is given to plants that are effectively known and used, and 1 is given to plants that are rarely mentioned; FC is the consensus among the informants. This value is obtained from the number of informants mentioning the species divided by the number of informants mentioning the most mentioned species.

The calculation of the UV and ICS considered the total mentions in the set of interviewees, and thereby, the UV and ICS of the species were obtained individually for each group. Moreover, the UV and ICS of the species mentioned by each group were obtained by considering only the remarks for the respective groups. To analyze whether the builder raftsmen tend to refer more to useful species than the non-builder raftsmen, we applied the Wilcoxon-Mann-Whitney nonparametric test. These statistical analyses were conducted after testing the normality of the data (Shapiro-Wilk) with the software R [45], *α* = 0.05.

## Results

### Socioeconomic characterization and knowledge related to fishing with a traditional raft

All interviewees were male with an average age of 50 years. Sixty-one percent of the subjects of the study were born where they currently live. Most of the raftsmen counted on fishing with a traditional raft as their primary income source, and many of them worked in other activities to complement or replace fishing. The most significant complementary occupations were farmer, bricklayer helper, servant, and carpenter. However, 10 individuals identified fishing as their only income source. Of the interviewed population of raftsmen, 27 were literate.

The average time of fishing activity with the traditional raft of the interviewed fishermen was 34 years. We observed that 25 of the raftsmen could build rafts. Moreover, we identified that the origin of the raftsmen’s knowledge was mostly related to shared experiences with older fishermen. There was a high frequency of positive responses (32 of those interviewed) about the transmission of this knowledge to other fishermen.

Regarding the changes perceived by these raftsmen, 33 alleged noticing some remarkable differences throughout the years in which they were fishing with a traditional raft. Among these, 31 and 24 classified these changes as adverse and positive, respectively. The most mentioned adverse changes were related to the reduction of fish (23 of those interviewed) and to the limits at the time on removing and transporting trees from the forest (18 of those interviewed). On the other hand, the most mentioned positive change was related to the introduction of the engine to the traditional raft (20 of those interviewed).

### Localization and characterization of the traditional rafts

**Fig. 2:**
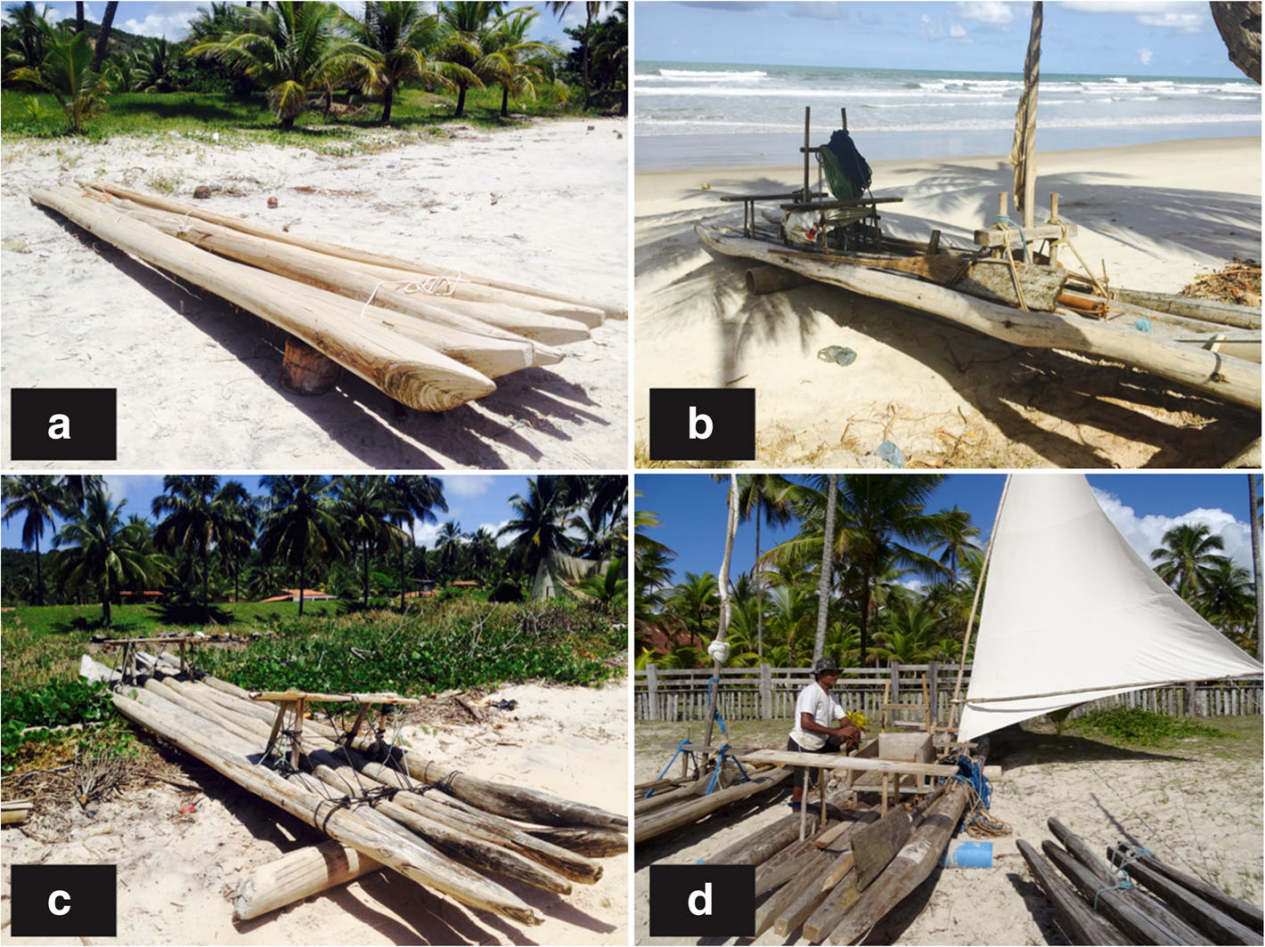
Traditional rafts found in Southern Bahia, Brazil. **a** Group 4 traditional raft. **b** Group 2 traditional raft. **c** Group 3 traditional raft. **d** Group 1 traditional raft

We identified traditional rafts made by the five groups of raftsmen, totaling 34 rafts, in which 20 were active rafts and 14 were inactive rafts (useless or in maintenance) (Fig. 2).

**Fig. 3:**
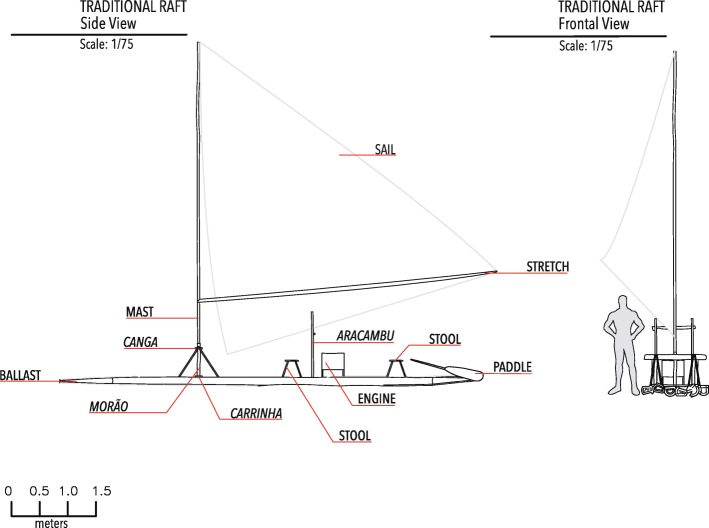
Technical drawing of the traditional raft used in Southern Bahia, Brazil, with all components in side and front view

Thirteen elements were identified, totaling 25 pieces for the composition of the traditional raft (Table 2; Fig. 3).

**Table 2:** Traditional raft components used in the South of Bahia (Brazil) with their functions, characteristics of wood allocated by the raftsmen to the species used in the production of these components

Components of the raft	Function	Wood characteristics
Ballast	Support all other components of the rafts and the crew on the surface of the water	Trunk: light, floating and rectilinear
Canga	Position the mast so that it remains vertically	Soft and should not break in splinters
Carrinha	Support for the mast	Tough in relation to weight
Morão	Position the canga and the van in parallel, enabling the sustaining of the mast	Resistant in relation to durability and rectilinear
Lathe	Used at the junction of the ballast logs	Resistant
Aracambu	Used to store nylon strings, baskets, baggage	Resistant in relation to durability and rectilinear
Stool	Seat in the pulp to the master and bow to the partner	Resistant in relation to durability
Stretch	Used to open and stretch the sail	Lightweight and rectilinear
Mast	Used to tie the sail cloth	Rectilinear, tough and flexible
Rod	Accessory used to go into and out of the sea	Light, floating and rectilinear
Hand paddle	Accessory used in the absence of the engine for the displacement of the boat	Any one that is not too heavy
Driving paddle	Accessory manipulated by the master of the raft. Used to steer or give direction for the displacement of the boat	Heavy

**Fig. 4:**
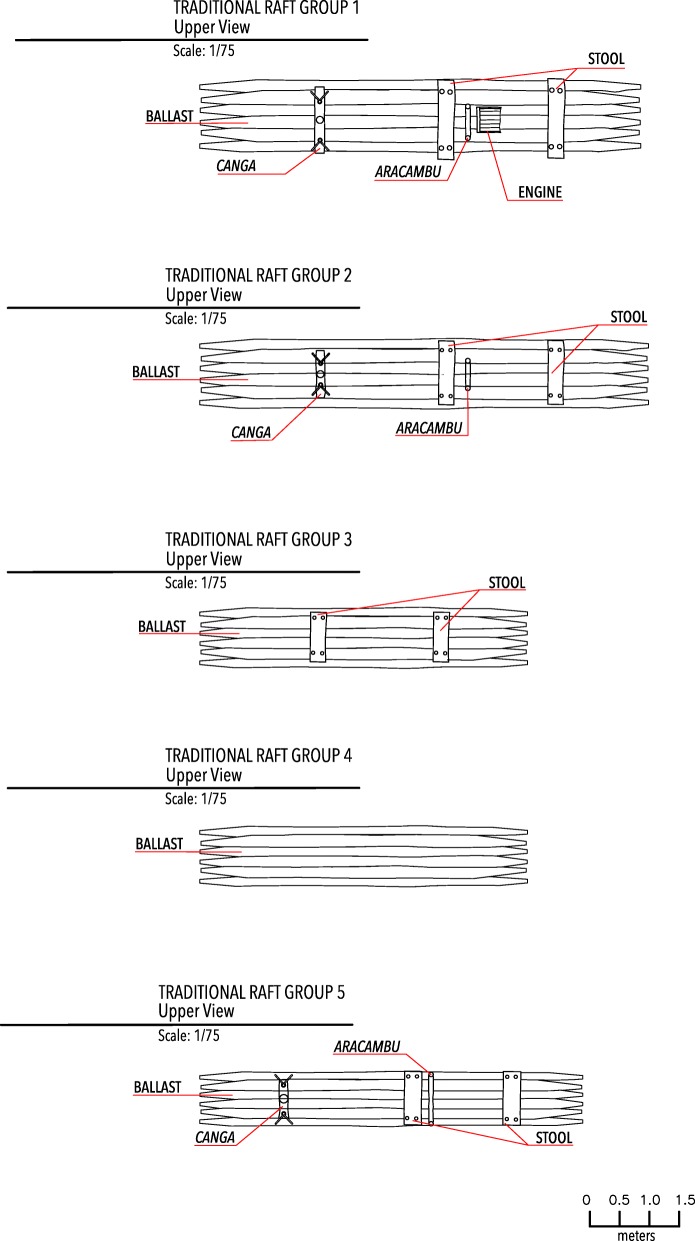
Technical drawing of the traditional raft models found in Southern Bahia, Brazil, with components in upper view

The raft of group 1 had ballast, a mast, and support devices, and one more component: the engine along with its bank. The raft of group 2 had ballast and support tools. Group 3 used rafts with ballast and stools as support devices. The raft of group 4 was simpler, being constructed only with ballast. The raft of group 5 was very similar to that of group 2, although it did not have the stretch component (Fig. 4).

The five groups of raftsmen presented differences related to the type of traditional raft used, how they fished, and average distance they traveled in the sea to reach the fishing boat or the fishing spot (Table 1).

### Usage of the species in the traditional raft in southern Bahia

We found 21 arboreal species that belonged to 17 families that are useful for the construction of the traditional raft (Table 3).

**Table 3:** Species cited as useful in building the traditional raft by rafters of Southern Bahia, Brazil

Family/species	Ethnospecies	Source	Citations	Uses/componets	Information dendrological [56–61]	U.V general	I.S.C. general
Anacardiaceae							
*Anacardium occidentale* L*.*	Cajueiro	N	41	*Canga*	Density 0.42 g/cm^3^. Lightweight, strong and long lasting	1.14	7.66
Annonaceae							
*Annona glabra* L*.*	Cortiça	N	20	*Canga*	Density 0.50 g/cm^3^	0.56	2.78
*Xylopia frutescens* Aubl*.*	Pindaíba	N	12	Mast, rod	Density 0.62 g/cm^3^. Moderately heavy, soft and easy to work, medium texture, medium hardiness and poor durability	0.33	1.11
Bignoniaceae							
*Tabebuia cassinoides (*Lam.) DC.	Taipóca	N	25	Stritch, hand paddle, rod	Density 0.37 g/cm^3^. Very light, glossy surface and read to the touch, fine texture, right grain, low mechanical resistance	0.69	13.33
Bombacaceae							
*Eriotheca macrophylla* (K. Schum.)	Embiruçu	N	1	Stool	**	0.03	0.03
Caryocaraceae							
*Caryocar brasiliense* Cambess. *	Pequi	N	21	*Canga*, *carrinha*, stool, hand paddle, driving paddle	Density 0.65 g/cm^3^. Moderately heavy, soft, strong and of low natural durability	0.58	4.44
Clusiaceae							
*Symphonia globulifera* L.f*.*	Alandi/Landirana	N	7	Mast, paddle, rod	**	0.19	0.78
Combretaceae							
*Conocarpus erectus* L. *	Mangue de Botão	N	4	*Morão*, *aracambu*, lathe	**	0.11	0.44
*Laguncularia racemosa* (L.) C.F.Gaertn	Mangue Branco/Mangue Manso	N	8	Stritch, mast, hand paddle, rod	**	0.22	0.69
Euphorbiaceae							
*Pogonophora schomburgkiana* Miers ex Benth.	Cocão	N	8	*Morão*, *aracambu*, mast	**	0.22	1.17
Fabaceae							
*Albizia polycephala* (Benth.) Killip	Muanza	N	28	Ballast	**	0.78	5.44
*Diplotropis incexis* Rizzini & A. Mattos *	Sucupira	N	11	*Carrinha*, driving paddle	**	0.30	0.50
Icacinaceae							
*Emmotum nitens* (Benth.) Miers	Aderno/Rouxinho	N	16	*Canga*, *carrinha*, *aracambu*, driving paddle	Density 0.93 g/cm^3^. Soft, thick texture, right grain, medium mechanical strength and low durability	0.44	2.92
Lauraceae							
*Aniba intermedia* (Meisn.) Mez	Louro	N	6	*Carrinha*, stool	**	0.17	0.42
Lecythidaceae							
*Eschweilera ovata* (Cambess.) Miers	Biriba	N	40	*Morão*, stool, *aracambu*, lathe, stritch, mast, *canga*	Density 1.03 g/cm^3^. Heavy, medium hardness, compact, uniform, tough and moderately durable	1.11	36.00
Malvaceae							
*Apeiba tibourbou* Aubl.	Pau de Jangada	N	36	Ballast	Very light, fluffy, from 0.12 to 0.30 g/cm^3^, soft and easy to work, low natural durability	1.00	8.00
Moraceae							
*Artocarpus heterophyllus* Lam*.*	Jaqueira	E	37	*Canga*, *carrinha*, *morão*, stool, driving paddle, hand paddle	Density 0.46 g/cm^3^	1.03	11.66
*Brosimum rubescens* Taub.	Conduru	N	28	Mast, stritch, rod	It has thick fibers and is tough and strong. Density 0.70 g/cm^3^	0.78	8.25
Pinaceae							
Pinus sp.	Pinho	E	16	Stool, stritch, mast	**	0.44	2.67
Rubiaceae							
*Genipa americana* L.	Jenipapo	N	10	Driving paddle, hand paddle	**	0.28	0.67
Sapotaceae							
*Manilkara maxima* T. D. Penn.	Massaranduba	N	28	*Carrinha*, driving paddle, hand paddle	**	0.78	7.94

*Species not collected

**Data not available

Raftsmen who built the rafts demonstrated significantly increased knowledge about the diversity of useful plants when compared to those who did not manufacture them (*W* = 210; *p* = 0.01246).

**Fig. 5:**
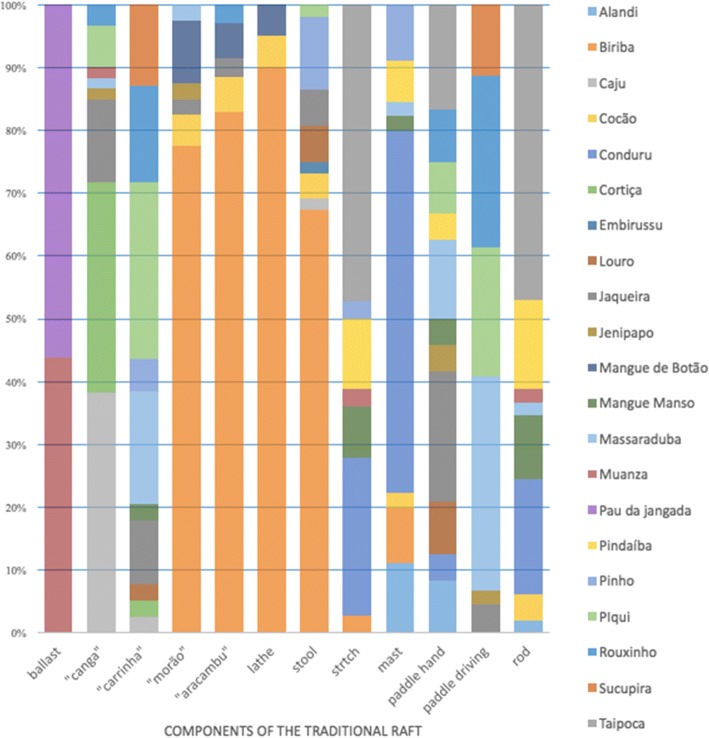
Species used in the making of the traditional raft’s components, South of Bahia, Brazil

Figure 5 highlights the species mentioned for the production of each component. Among the elements, the ballast, mast, lathe, *aracambu*, stool, and *morão* were made of just one preferred species. The *canga*, *carrinha*, hand paddle, and driving paddle components were composed of more than two preferential species. Figure 5 also highlights that *biriba* is the preferential species for the production of four elements, the *morão*, *aracambu*, lathe and stool, and that the *pau de jangada*, in addition to being the preferred species for ballast construction, is also exclusively used to produce this component.

**Fig. 6:**
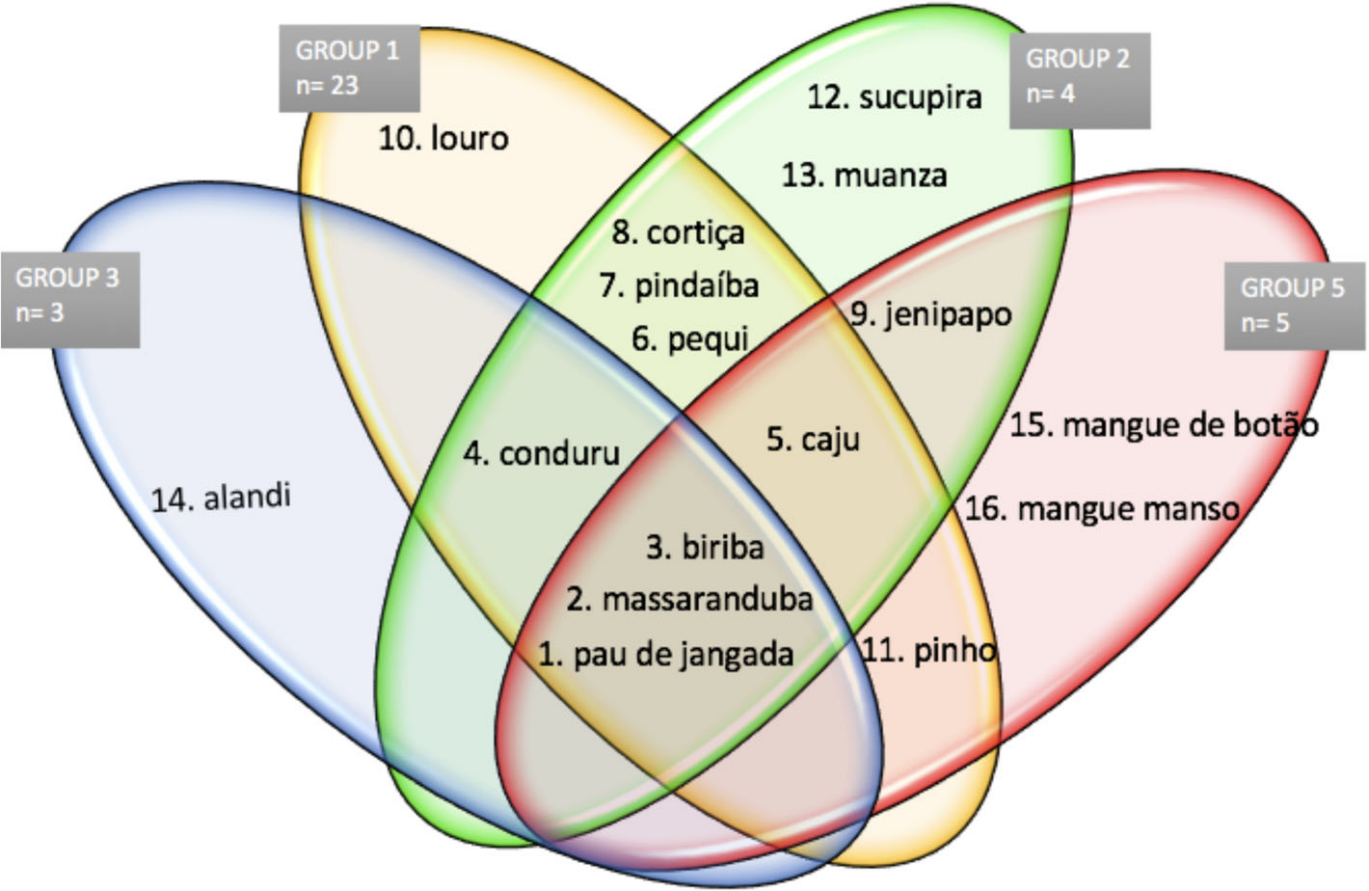
Venn’s diagram representing the preferred species in the traditional raft building for each of the groups analyzed in South of Bahia, Brazil. 1-*Apeiba tibourbou*, 2-*Manilkara maxima*, 3-*Eschweilera ovata*, 4-*Brosimum rubescens*, 5-*Anacardium occidentale*, 6-*Caryocar brasiliense*, 7-*Xylopia frutescens*, 8-*Annona glabra*, 9-*Genipa americana*, 10-*Aniba intermedia*, 11-*Pinus* sp*.*, 12-*Diplotropis incexis*, 13-*Albizia polycephala*, 14-*Symphonia globulifera*, 15-*Conocarpus erectus*, and 16-*Laguncularia racemosa*

The criteria to choose the species used in raft construction are associated with the functions of the components, and these factors are directly related to the features of the wood of the species (Table 2). The preferential species were determined for the set of interviewed raftsmen and individually for each of the groups of raftsmen (1, 2, 3, and 5) (Fig. 6). Moreover, we observed that only three species were preferential for all groups to produce the same component: *massaranduba* to produce the mast, *pau de jangada* to produce the ballast, and *biriba* to produce lathes and stools.

Using a Venn diagram (Fig. 6), we identified that there are preferential species for each group: *louro* for group 1, *sucupira* and *muanza* for group 2, *alandi* for group 3, and *mangue manso* and *mangue de botão* for group 5. It is noticeable that groups 1 and 2 have the most substantial number of common preferential species and that there are three common preferred species for all the analyzed groups.

Additionally, we observed that *biriba* had a higher UV value for both the set of interviewees and for the groups. Of the 21 species used in the construction of the traditional raft for all the interviewees, 19.05% showed UVs higher or equal to 1: *taipóca*, *biriba*, *conduru*, and *pau de jangada*. Species having UVs between 0.58 and 0.83 represented 33.33% of the species. The most significant percentage (47.6%) represented species with UVs between 0.08 and 0.50.

Regarding the ICS results, the highest percentage (66.66%) represented species that obtained values higher than 1. The species that had ICS values between 0.50 and 0.78 represented 19.05% of the species. Only 14.28% of the species had ICS values lower than 0.50. Like the UV result, the more prominent ICS value was observed in *biriba* for both the set of interviewees and for the groups (Table 3).

## Discussion

### General aspects of the raft and the raftsmen

Fishing with a traditional raft by raftsmen in southern Bahia is mostly practised by men and is becoming increasingly scarce along the northeast coast [21, 46]. Despite the existence of active spots for traditional raft fishing in this region, we observed a small number of fishermen joining this raft population (Table 1).

Raft fishing and building techniques were learned from the life experiences of older fishermen without any kinship required. The way that knowledge is transmitted between the interviewed fishermen is different compared to other artisanal fishing communities, where knowledge is transmitted throughout the family circle [18, 46, 47]. The knowledge shared between the raftsmen is associated with the number of raftsmen building the rafts. This result contrasts with those found for groups of handcrafted canoe builders in the state of *Piauí*, corresponding to nearly 7% of all the fishing community [17]. Throughout the years of experience, these fishermen have actively transmitted their knowledge to other generations [22] by teaching new apprentices.

The interviewed raftsmen population practises artisanal fishing, which is the income for nearly 25 of the population regarding both the sale and the consumption of fish. The raftsmen have been politically and socially active throughout the history of the country and are aware of the needs and difficulties that face the development of their daily practices [48, 49]. The studied population revealed itself to be perceptive to the changes that occurred and influenced the practices related to the traditional raft. Like the artisanal fishing community from *Carne de Vaca* beach in the state of Pernambuco [50], 83% of the changes with positive influence on the raftsmen interviewed in this study were associated with the adaptability of the watercraft compared to other motored watercrafts. However, the changes with negative influence on their practices were more noticeable and represented the opinion of 31 of the raftsmen. The most significant complaint was associated with the reduction of the fishing stocks. Raftsmen reported that there was increasing competition with large vessels next to the fishing spots. Another negative aspect was related to the difficulty of obtaining the necessary natural resources to construct the traditional raft due to inspection at the time of extraction or deforestation. These two factors were also reported in other communities as being among the main factors limiting the construction of artisanal watercrafts [2, 12, 17, 50]. Some authors [21] believe that the reduced use of the traditional raft in the northeast Brazilian region is mostly related to the lack of plant resources necessary for its construction. However, given the presented results, it is evident that there are many factors that can contribute to the reduction and replacement of the traditional raft by other types of watercrafts. According to the National Historic and Artistic Heritage Institute—IPHAN (2011), the traditional Brazilian raft is a watercraft threatened by extinction.

The disappearance of traditional watercrafts with cultural importance to the local communities has already been reported in studies developed in Peru and in Polynesia, where authors highlighted that the association between culture and the environment is strengthened with the traditional practices of the communities [12, 13]. Therefore, the disappearance of a watercraft that was kept for such practices consequently leads to the alienation of these local communities from the environment and the natural resources surrounding them.

### Use of the species in the traditional raft in southern Bahia

To analyze how plant species are used in the traditional raft, an understanding of how this watercraft is built and the models found along the seacoast studied is necessary.

The traditional raft is derived from a simple indigenous watercraft formed by joining wooden logs with liana ties (ballast) and is used by indigenous populations mostly for river fishing [51]. After some structural changes influenced by Portuguese traditions, this watercraft became able to navigate in the high sea. Therefore, the traditional raft described by Cascudo [46] consists not only of the ballast but also of a structure to support the sail and at least two fishermen on the open sea. Models of the traditional raft observed along the studied area varied according to the groups of fishermen found. The models made by groups 1, 2, and 5 were similar to the rafts described in the literature, whereas the models of groups 3 and 4 were more similar to the watercrafts used by the indigenous population before Brazil’s colonization (Fig. 4). The structural differences between the rafts found can be explained by the type of fishing chosen by the groups of raftsmen. Raftsmen from groups 1, 2, and 5 practise line fishing, so they need rafts with a sail to travel longer distances until they find fishing spots. On the other hand, groups 3, 4, and 5 practised trawling fishing, which dismisses the sail because there is no need to get far from the coast. However, to make it possible for this type of fishing to occur, it is necessary for a large continental platform to exist [30] at the spot chosen to displace the trawls. Only groups 3, 4, and 5 were favorably located to practise this type of fishing. Another adaptation observed in the raft built by group 1 was the introduction of a small engine previously used in the old flour mills (interview 18). The introduction of the engine might explain, for instance, the difference in the number of raftsmen in group 1 compared to the number of raftsmen in the other groups. The engine facilitates the activity by reducing the time spent arriving at the fishing spots by applying less effort to displace the raft and by increasing the possibility of exploring new fishing spots. In addition, the engine keeps construction and maintenance cost of the raft low compared to other motorized watercrafts. Therefore, the different models of traditional raft found are the result of each group’s needs.

In addition, all of the species occupy the technological category of use because their wood is used to produce the components of the watercraft. Moreover, this category represents the species that endure manipulation of their raw material to create useful elements (tools, furniture, watercrafts) [52]. Those pieces are individually made by the raftsmen who choose an arboreal species with a specific wood to produce each component. Thus, if some component, for instance, the *carrinha*, needs to be resistant enough to support the weight of the mast and the sailcloth, the raftsmen choose a species with wood that is resistant to this weight, popularly known as *fixe* wood. This specificity explains the diversity of species used to construct the traditional raft. The same chosen criteria for the usage of the species were observed in watercrafts built by fishermen on the Mediterranean western coast in Italy, in the construction of a canoe in Pohnpei, in the Federated States of Micronesia, and in the construction of a canoe on the island of Kabara in Polynesia. The authors had similar observations when compared to those in this study regarding the diversity of species used: 25 useful species in Italy, 27 in Micronesia, and 20 in Polynesia [2, 11, 13].

Altogether, we identified 21 species that are useful for constructing the traditional raft, but not all of them together compose the same watercraft. This selection occurs because the raftsmen have more than one species that can be applied to produce each component. In this way, it is expected that the specialists mention a more significant diversity of useful species in the raft because the raftsmen are aware of the similarity among species. In this way, the *carrinha* of a raft can be made of the *pequi*, *massaranduba* (cow-tree), *roxinho* (purple wood/purpleheart), *sucupira*, or jack tree wood. All of these wood types are recognized by the raftsmen as ideal to make this component because they have *fixe* wood. Therefore, each species cataloged is useful for a type of element in the raft, with the variation depending on the function that this component has in the raft and on the morpho-anatomical characteristics of the wood of each species. Consequently, each feature corresponds to a quantity of species capable of being used for its construction. Nevertheless, we detected that the same species have more frequent mentions than others concerning the production of some elements and that the number of useful species varies according to the created feature (Fig. 5). By calculating the average number of mentions of the valuable species needed to produce each part, it is possible to determine the favorite species for each component.

The determination of the favorite species by the set of raftsmen studied was performed through the analysis of the mentions from all interviewees. This analysis was conducted with each group by considering only the mentions of the group. The observation by the group revealed that the favorite species of that population was very biased towards the species chosen by group 1. This observation likely occurred because group 1 had a more substantial number of raftsmen compared to other groups. Figure 6 shows the favorite species of each group that are uncommon between the groups and that some groups share more favorite species than others. Both situations may be related to the location of the groups, either because they are spatially close, as in groups 1 and 2, which share the same preferences, or because they are close to areas that favor the use of some species. This last observation was found in group 5, which is located near mangrove forest areas and selected two mangrove plant species as their favorite. It can also be noted that only 3 out of 21 species used by raftsmen are preferential to all the groups.

Based on the number of species used to make each component and on the frequency with which the species were mentioned, we observed that *biriba* and *pau de jangada* were the most frequently used. *Biriba* was mentioned by all the raftsmen and was defined as being valuable for more than one component, which was observed by a higher UV in the raft and a higher ICS. This species was also the favorite for producing components (*morão*, lathe, *aracambu*, and stool). The diversity and frequency of usage of the *biriba* wood in the raft may be associated with its ‘plastic’ attribute—it is useful for more than one component, it is highly available, and it is practical to use. A raftsman declared that he found it “ready to use, with the right ‘gauge’” and that “one can find *biriba* just by the roadside” (interview 28). On the other hand, *pau de jangada* wood was remarkably the species used exclusively for the ballast, with only *muanza* as a backup to make this component. However, the use of *muanza* in the raft is practised by groups 1, 2, and 3, and it is evident that the construction of the traditional raft by groups 4 and 5 would be impossible if the *pau de jangada* was not available. This information indicates that the existence of the traditional raft is directly associated with the availability of such species. Caruso [53] reported this in an interview with a raftsman: “Nowadays, unfortunately, this type of raft (six logs *piúba* wooden raft) does not exist anymore because the extraction of the tree has been forbidden. Now, you can only see the *piúba* raft in the museum”. This affirmation is confirmed by the fact that one of the main components of the watercraft depends on the species *pau de jangada*.

By considering the UV of the species, more than half presented low values (< 1.00). By examining the ICS, we observed the opposite (Table 3). This result indicates that the ICS represented a notable usage for these species in the traditional raft because its calculation includes not only the variables related to the type of usage of the species but also the frequency of usage, the preference for the species to the detriment of others available, and if there is an interaction, besides the extraction, between the species used and the fishermen. As a result, the species *pau de jangada*, *taipóca*, *muanza*, and *cajueiro* were better represented by the ICS than by the UV, which is mainly determined by the different uses of those species in the traditional raft.

Given the cultural richness present in the traditional raft, the extensive knowledge obtained by the raftsmen concerning the natural resources they use, the native biodiversity of the Atlantic Forest, and the synergic interaction between these elements, the need to establish joint actions that guarantee their dynamics becomes evident. Moreover, the sites where people still practise actions that directly connect the survival of human groups to the use of natural resources may also be maintained. This direct relationship can be the key to making social groups feel like they are part of the environment due to their direct responsibility for preserving this area and consequently perpetuating their practices. Then, the imposing measures that are taken should be reduced in such a way that the existence of areas with rich biodiversity, such as the Atlantic Forest, can be guaranteed. Currently, the role of areas of protection [31] and of ongoing legislation to protect the species of the Atlantic Forest [54, 55] is essential to the existence of this fragile, naturally priceless biome. The recognition of the construction of the raft as a cultural heritage of the region could allow the permanence of the existing construction practice, even with the current laws of protection of biodiversity [31, 55] and the current change of fishing technology to synthetic materials. The permanence of this practice could maintain diverse relationships with the surrounding environment, allowing for even greater success in the conservation of resources.

In several places in the world and in Brazil, there has been a substitution of natural resources for synthetic material, as described by Rodón et al. [12]. However, in the existence of a public policy that recognizes traditional knowledge and guarantees, if it is of local interest, its permanence would allow the perpetuation of local knowledge.

The studied area is an example of a location with biological and cultural diversity, where strategies that involve both aspects can be developed to strengthen the local cultural identity and to guarantee the execution of traditional practices that would only be possible with local communities taking responsibility concerning the usage of the natural resources available.

## Conclusions

The raft-related population from southern Bahia is aware of the arboreal plant species connected to their fishing activity. The primary association between the fishermen and the species they use appears in the traditional practice of building the raft, which is done by the raftsmen themselves.

Due to the high sophistication level in the construction of this watercraft, raftsmen need a more profound knowledge of the morpho-anatomical characteristics of the woods in the species used, which can guarantee the efficiency of the traditional raft in fishing-related activities. The models of the traditional rafts result from the necessities of the groups from each location. Moreover, the practicality and functionality of the watercraft in the face of the adversities found by the raft community are also remarkable. We identified the preferential usage of some species over others. *Biriba* wood has been found to be valuable for building such watercraft, and the *pau de jangada* wood was the only irreplaceable, or nearly irreplaceable, species in the production of the ballast of the raft.

Faced with the need for the plant resources required for the practices of this population, developing strategies that combine the conservation of the natural resources available with the preservation of the local culture, while respecting the interrelation between “man-plant-sea,” is essential.

## Abbreviations

CEPEC: Cocoa Research Center
HUESC: Herbarium of the State University of Santa Cruz
IPHAN: National Historic and Artistic Heritage Institute
